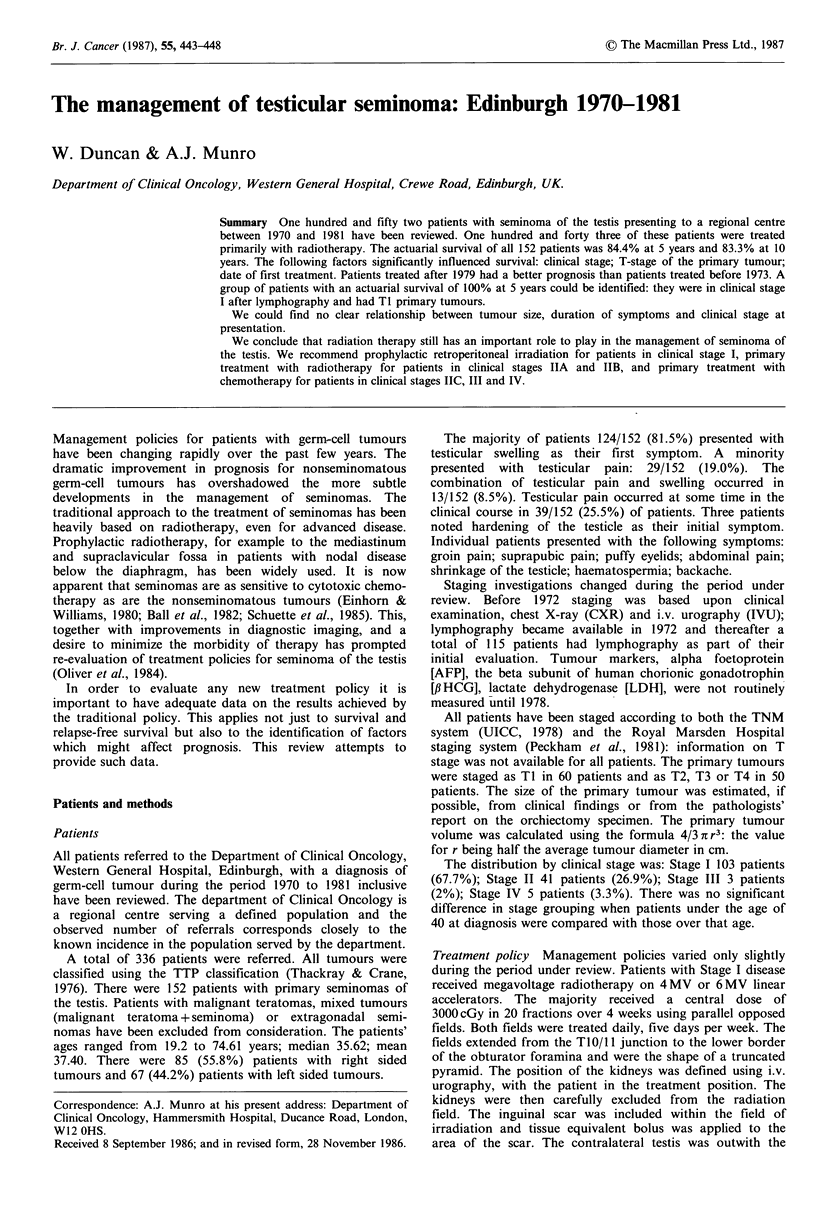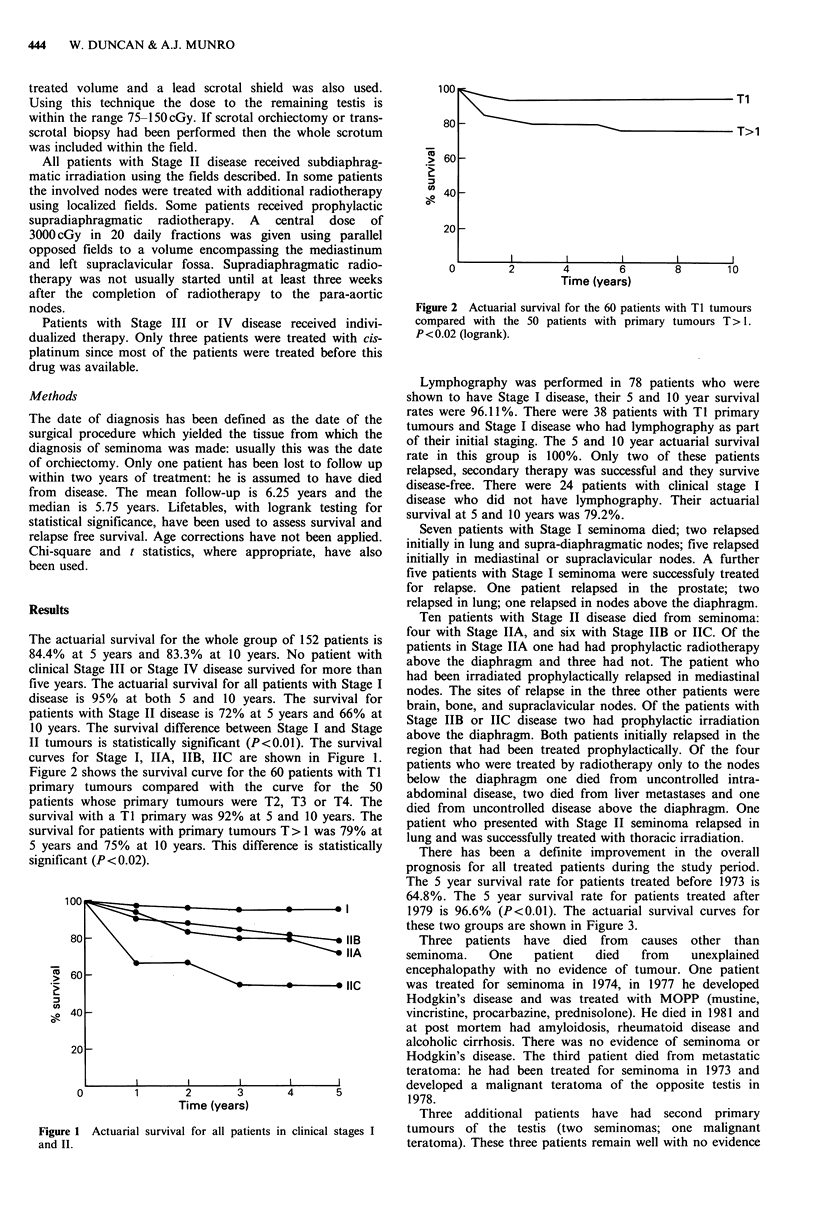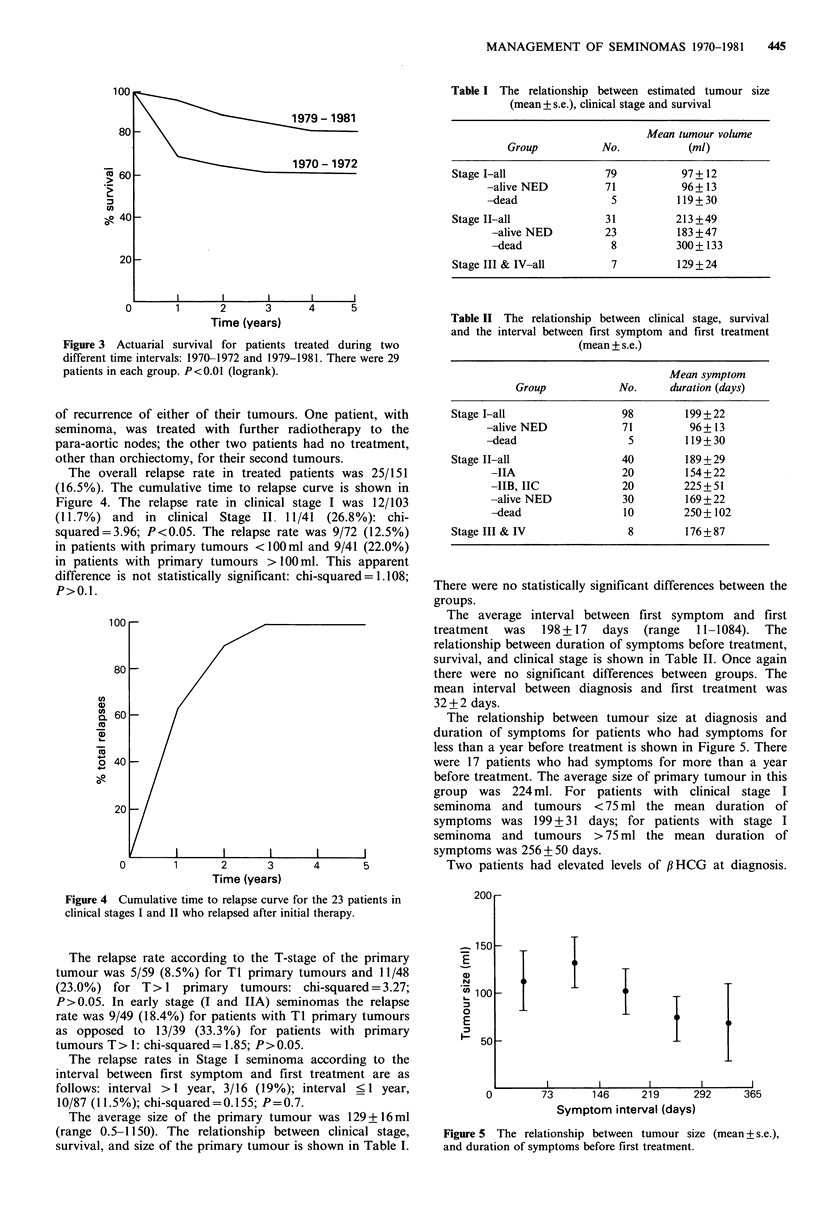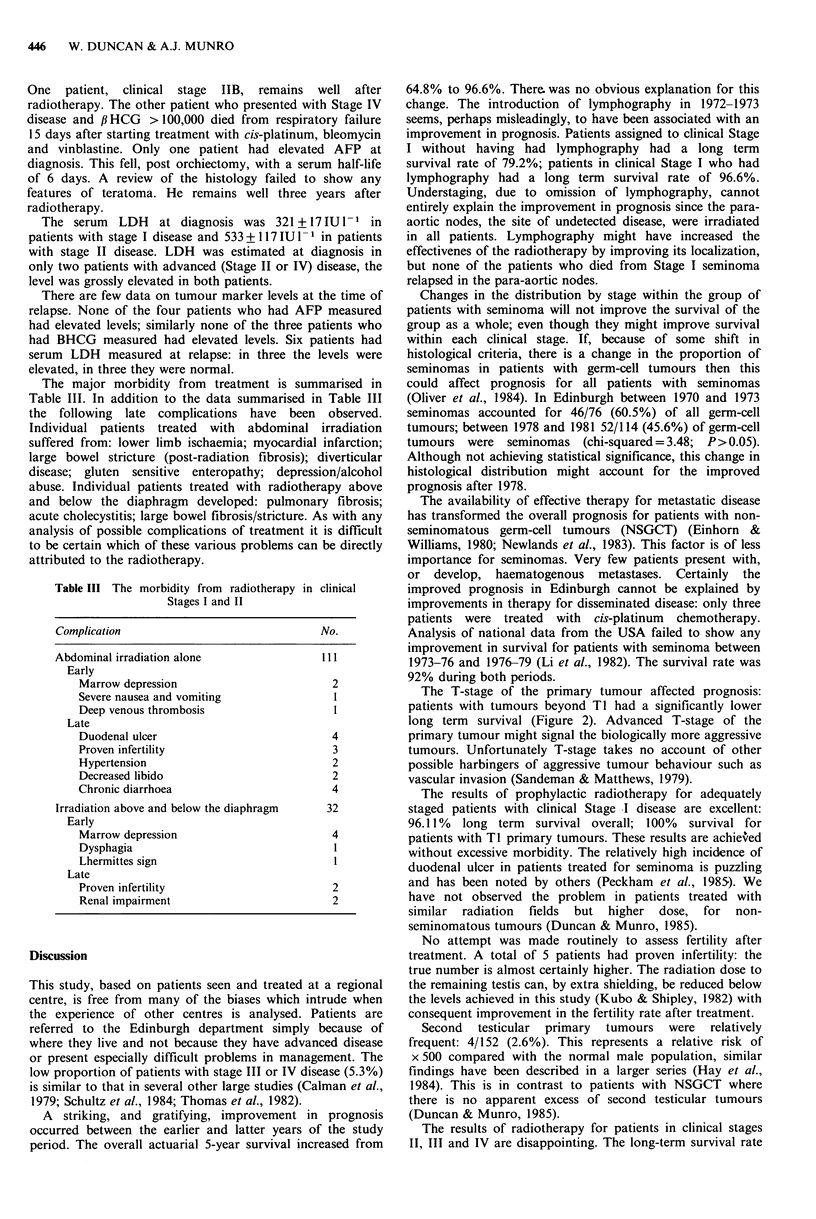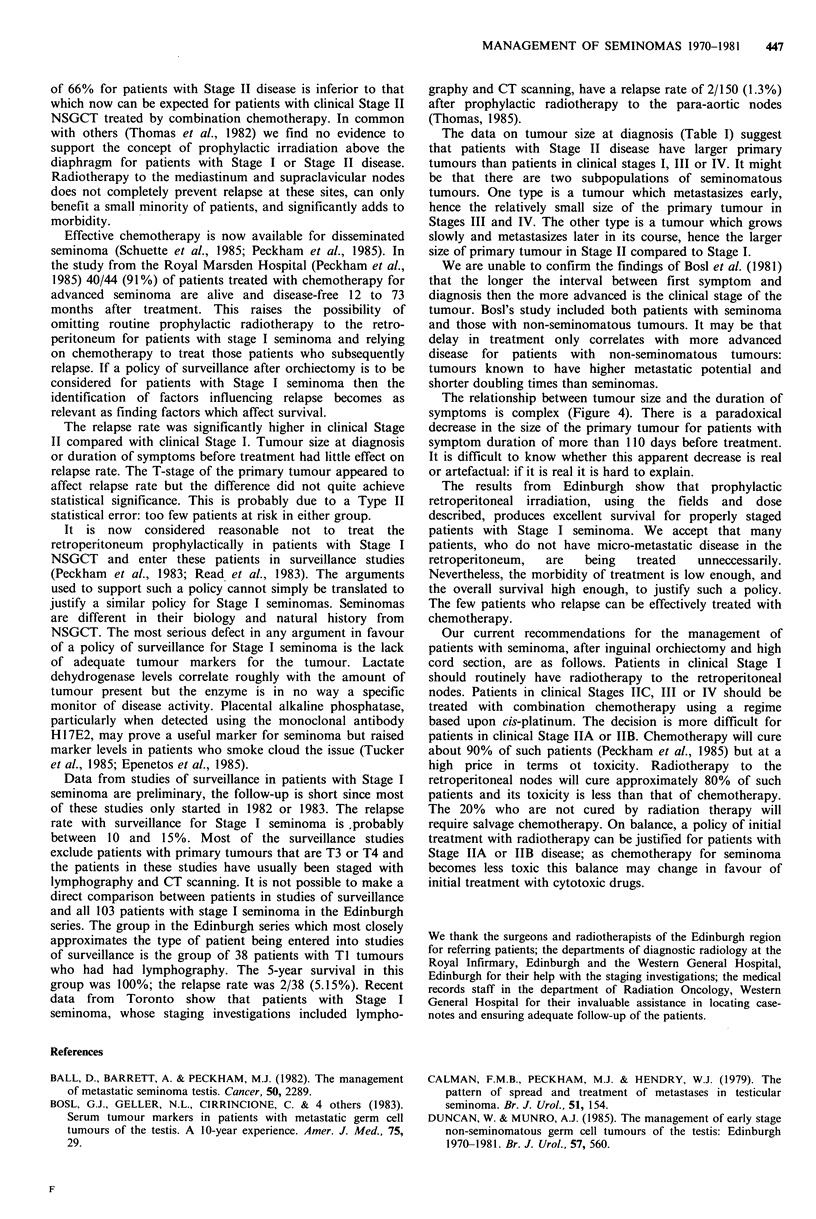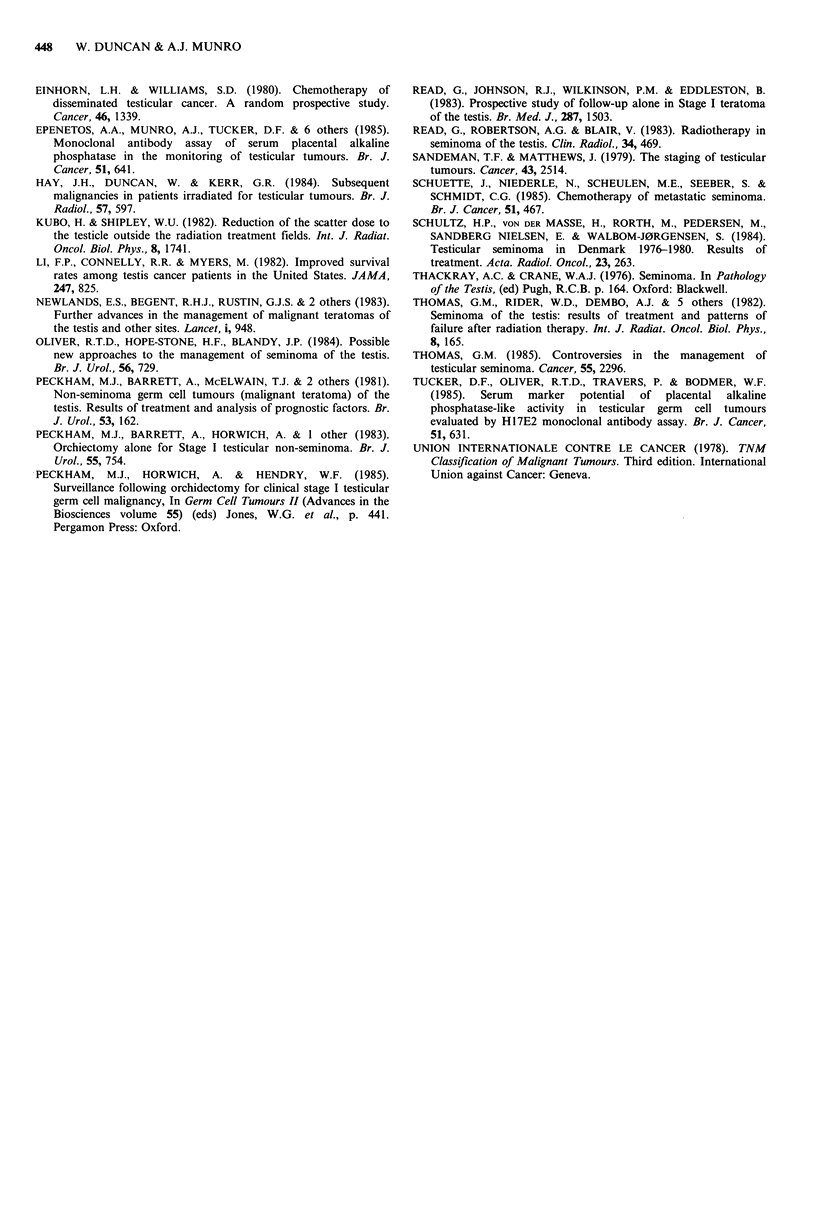# The management of testicular seminoma: Edinburgh 1970-1981.

**DOI:** 10.1038/bjc.1987.87

**Published:** 1987-04

**Authors:** W. Duncan, A. J. Munro

## Abstract

One hundred and fifty two patients with seminoma of the testis presenting to a regional centre between 1970 and 1981 have been reviewed. One hundred and forty three of these patients were treated primarily with radiotherapy. The actuarial survival of all 152 patients was 84.4% at 5 years and 83.3% at 10 years. The following factors significantly influenced survival: clinical stage; T-stage of the primary tumour; date of first treatment. Patients treated after 1979 had a better prognosis than patients treated before 1973. A group of patients with an actuarial survival of 100% at 5 years could be identified: they were in clinical stage I after lymphography and had T1 primary tumours. We could find no clear relationship between tumour size, duration of symptoms and clinical stage at presentation. We conclude that radiation therapy still has an important role to play in the management of seminoma of the testis. We recommend prophylactic retroperitoneal irradiation for patients in clinical stage I, primary treatment with radiotherapy for patients in clinical stages IIA and IIB, and primary treatment with chemotherapy for patients in clinical stages IIC, III and IV.


*Br. J. Cancer* (1987), **55**, 443–448 © The Macmillan Press Ltd., 1987

### The management of testicular seminoma: Edinburgh 1970–1981

W. Duncan & A.J. Munro

*Department of Clinical Oncology, Western General Hospital, Crewe Road, Edinburgh, UK.*

**Summary** One hundred and fifty two patients with seminoma of the testis presenting to a regional centre between 1970 and 1981 have been reviewed. One hundred and forty three of these patients were treated primarily with radiotherapy. The actuarial survival of all 152 patients was 84.4% at 5 years and 83.3% at 10 years. The following factors significantly influenced survival: clinical stage; T-stage of the primary tumour; date of first treatment. Patients treated after 1979 had a better prognosis than patients treated before 1973. A group of patients with an actuarial survival of 100% at 5 years could be identified: they were in clinical stage I after lymphography and had T1 primary tumours.

We could find no clear relationship between tumour size, duration of symptoms and clinical stage at presentation.

We conclude that radiation therapy still has an important role to play in the management of seminoma of the testis. We recommend prophylactic retroperitoneal irradiation for patients in clinical stage I, primary treatment with radiotherapy for patients in clinical stages IIA and IIB, and primary treatment with chemotherapy for patients in clinical stages IIC, III and IV.

Management policies for patients with germ-cell tumours have been changing rapidly over the past few years. The dramatic improvement in prognosis for nonseminomatous germ-cell tumours has overshadowed the more subtle developments in the management of seminomas. The traditional approach to the treatment of seminomas has been heavily based on radiotherapy, even for advanced disease. Prophylactic radiotherapy, for example to the mediastinum and supraclavicular fossa in patients with nodal disease below the diaphragm, has been widely used. It is now apparent that seminomas are as sensitive to cytotoxic chemotherapy as are the nonseminomatous tumours (Einhorn & Williams, 1980; Ball *et al.*, 1982; Schuette *et al.*, 1985). This, together with improvements in diagnostic imaging, and a desire to minimize the morbidity of therapy has prompted re-evaluation of treatment policies for seminoma of the testis (Oliver *et al.*, 1984).

In order to evaluate any new treatment policy it is important to have adequate data on the results achieved by the traditional policy. This applies not just to survival and relapse-free survival but also to the identification of factors which might affect prognosis. This review attempts to provide such data.

### Patients and methods

### *Patients*

All patients referred to the Department of Clinical Oncology, Western General Hospital, Edinburgh, with a diagnosis of germ-cell tumour during the period 1970 to 1981 inclusive have been reviewed. The department of Clinical Oncology is a regional centre serving a defined population and the observed number of referrals corresponds closely to the known incidence in the population served by the department.

A total of 336 patients were referred. All tumours were classified using the TTP classification (Thackray & Crane, 1976). There were 152 patients with primary seminomas of the testis. Patients with malignant teratomas, mixed tumours (malignant teratoma+seminoma) or extragonadal seminomas have been excluded from consideration. The patients' ages ranged from 19.2 to 74.61 years; median 35.62; mean 37.40. There were 85 (55.8%) patients with right sided tumours and 67 (44.2%) patients with left sided tumours.

Correspondence: A.J. Munro at his present address: Department of Clinical Oncology, Hammersmith Hospital, Ducance Road, London, W12 0HS.

Received 8 September 1986; and in revised form, 28 November 1986.

The majority of patients 124/152 (81.5%) presented with testicular swelling as their first symptom. A minority presented with testicular pain: 29/152 (19.0%). The combination of testicular pain and swelling occurred in 13/152 (8.5%). Testicular pain occurred at some time in the clinical course in 39/152 (25.5%) of patients. Three patients noted hardening of the testicle as their initial symptom. Individual patients presented with the following symptoms: groin pain; suprapubic pain; puffy eyelids; abdominal pain; shrinkage of the testicle; haematospermia; backache.

Staging investigations changed during the period under review. Before 1972 staging was based upon clinical examination, chest X-ray (CXR) and i.v. urography (IVU); lymphography became available in 1972 and thereafter a total of 115 patients had lymphography as part of their initial evaluation. Tumour markers, alpha foetoprotein [AFP], the beta subunit of human chorionic gonadotrophin [$\beta$HCG], lactate dehydrogenase [LDH], were not routinely measured until 1978.

All patients have been staged according to both the TNM system (UICC, 1978) and the Royal Marsden Hospital staging system (Peckham *et al.*, 1981): information on T stage was not available for all patients. The primary tumours were staged as T1 in 60 patients and as T2, T3 or T4 in 50 patients. The size of the primary tumour was estimated, if possible, from clinical findings or from the pathologists' report on the orchiectomy specimen. The primary tumour volume was calculated using the formula $4/3\pi r^3$: the value for $r$ being half the average tumour diameter in cm.

The distribution by clinical stage was: Stage I 103 patients (67.7%); Stage II 41 patients (26.9%); Stage III 3 patients (2%); Stage IV 5 patients (3.3%). There was no significant difference in stage grouping when patients under the age of 40 at diagnosis were compared with those over that age.

*Treatment policy* Management policies varied only slightly during the period under review. Patients with Stage I disease received megavoltage radiotherapy on 4 MV or 6 MV linear accelerators. The majority received a central dose of 3000 cGy in 20 fractions over 4 weeks using parallel opposed fields. Both fields were treated daily, five days per week. The fields extended from the T10/11 junction to the lower border of the obturator foramina and were the shape of a truncated pyramid. The position of the kidneys was defined using i.v. urography, with the patient in the treatment position. The kidneys were then carefully excluded from the radiation field. The inguinal scar was included within the field of irradiation and tissue equivalent bolus was applied to the area of the scar. The contralateral testis was outwith the treated volume and a lead scrotal shield was also used. Using this technique the dose to the remaining testis is within the range 75–150 cGy. If scrotal orchiectomy or trans-scrotal biopsy had been performed then the whole scrotum was included within the field.

All patients with Stage II disease received subdiaphragmatic irradiation using the fields described. In some patients the involved nodes were treated with additional radiotherapy using localized fields. Some patients received prophylactic supradiaphragmatic radiotherapy. A central dose of 3000 cGy in 20 daily fractions was given using parallel opposed fields to a volume encompassing the mediastinum and left supraclavicular fossa. Supradiaphragmatic radiotherapy was not usually started until at least three weeks after the completion of radiotherapy to the para-aortic nodes.

Patients with Stage III or IV disease received individualized therapy. Only three patients were treated with *cis*-platinum since most of the patients were treated before this drug was available.

*Methods*

The date of diagnosis has been defined as the date of the surgical procedure which yielded the tissue from which the diagnosis of seminoma was made: usually this was the date of orchiectomy. Only one patient has been lost to follow up within two years of treatment: he is assumed to have died from disease. The mean follow-up is 6.25 years and the median is 5.75 years. Lifetables, with logrank testing for statistical significance, have been used to assess survival and relapse free survival. Age corrections have not been applied. Chi-square and *t* statistics, where appropriate, have also been used.

### Results

The actuarial survival for the whole group of 152 patients is 84.4% at 5 years and 83.3% at 10 years. No patient with clinical Stage III or Stage IV disease survived for more than five years. The actuarial survival for all patients with Stage I disease is 95% at both 5 and 10 years. The survival for patients with Stage II disease is 72% at 5 years and 66% at 10 years. The survival difference between Stage I and Stage II tumours is statistically significant ($P<0.01$). The survival curves for Stage I, IIA, IIB, IIC are shown in Figure 1. Figure 2 shows the survival curve for the 60 patients with T1 primary tumours compared with the curve for the 50 patients whose primary tumours were T2, T3 or T4. The survival with a T1 primary was 92% at 5 and 10 years. The survival for patients with primary tumours T>1 was 79% at 5 years and 75% at 10 years. This difference is statistically significant ($P<0.02$).

**Figure 1** Actuarial survival for all patients in clinical stages I and II.

**Figure 2** Actuarial survival for the 60 patients with T1 tumours compared with the 50 patients with primary tumours T>1. $P<0.02$ (logrank).

Lymphography was performed in 78 patients who were shown to have Stage I disease, their 5 and 10 year survival rates were 96.11%. There were 38 patients with T1 primary tumours and Stage I disease who had lymphography as part of their initial staging. The 5 and 10 year actuarial survival rate in this group is 100%. Only two of these patients relapsed, secondary therapy was successful and they survive disease-free. There were 24 patients with clinical stage I disease who did not have lymphography. Their actuarial survival at 5 and 10 years was 79.2%.

Seven patients with Stage I seminoma died; two relapsed initially in lung and supra-diaphragmatic nodes; five relapsed initially in mediastinal or supraclavicular nodes. A further five patients with Stage I seminoma were successfuly treated for relapse. One patient relapsed in the prostate; two relapsed in lung; one relapsed in nodes above the diaphragm.

Ten patients with Stage II disease died from seminoma: four with Stage IIA, and six with Stage IIB or IIC. Of the patients in Stage IIA one had had prophylactic radiotherapy above the diaphragm and three had not. The patient who had been irradiated prophylactically relapsed in mediastinal nodes. The sites of relapse in the three other patients were brain, bone, and supraclavicular nodes. Of the patients with Stage IIB or IIC disease two had prophylactic irradiation above the diaphragm. Both patients initially relapsed in the region that had been treated prophylactically. Of the four patients who were treated by radiotherapy only to the nodes below the diaphragm one died from uncontrolled intra-abdominal disease, two died from liver metastases and one died from uncontrolled disease above the diaphragm. One patient who presented with Stage II seminoma relapsed in lung and was successfully treated with thoracic irradiation.

There has been a definite improvement in the overall prognosis for all treated patients during the study period. The 5 year survival rate for patients treated before 1973 is 64.8%. The 5 year survival rate for patients treated after 1979 is 96.6% ($P<0.01$). The actuarial survival curves for these two groups are shown in Figure 3.

Three patients have died from causes other than seminoma. One patient died from unexplained encephalopathy with no evidence of tumour. One patient was treated for seminoma in 1974, in 1977 he developed Hodgkin's disease and was treated with MOPP (mustine, vincristine, procarbazine, prednisolone). He died in 1981 and at post mortem had amyloidosis, rheumatoid disease and alcoholic cirrhosis. There was no evidence of seminoma or Hodgkin's disease. The third patient died from metastatic teratoma: he had been treated for seminoma in 1973 and developed a malignant teratoma of the opposite testis in 1978.

Three additional patients have had second primary tumours of the testis (two seminomas; one malignant teratoma). These three patients remain well with no evidence

**Figure 3** Actuarial survival for patients treated during two different time intervals: 1970–1972 and 1979–1981. There were 29 patients in each group. $P<0.01$ (logrank).

of recurrence of either of their tumours. One patient, with seminoma, was treated with further radiotherapy to the para-aortic nodes; the other two patients had no treatment, other than orchiectomy, for their second tumours.

The overall relapse rate in treated patients was 25/151 (16.5%). The cumulative time to relapse curve is shown in Figure 4. The relapse rate in clinical stage I was 12/103 (11.7%) and in clinical Stage II. 11/41 (26.8%): chi-squared=3.96; $P<0.05$. The relapse rate was 9/72 (12.5%) in patients with primary tumours <100 ml and 9/41 (22.0%) in patients with primary tumours >100 ml. This apparent difference is not statistically significant: chi-squared=1.108; $P>0.1$.

**Figure 4** Cumulative time to relapse curve for the 23 patients in clinical stages I and II who relapsed after initial therapy.

The relapse rate according to the T-stage of the primary tumour was 5/59 (8.5%) for T1 primary tumours and 11/48 (23.0%) for T>1 primary tumours: chi-squared=3.27; $P>0.05$. In early stage (I and IIA) seminomas the relapse rate was 9/49 (18.4%) for patients with T1 primary tumours as opposed to 13/39 (33.3%) for patients with primary tumours T>1: chi-squared=1.85; $P>0.05$.

The relapse rates in Stage I seminoma according to the interval between first symptom and first treatment are as follows: interval >1 year, 3/16 (19%); interval ≦1 year, 10/87 (11.5%); chi-squared=0.155; $P=0.7$.

The average size of the primary tumour was 129±16 ml (range 0.5–1150). The relationship between clinical stage, survival, and size of the primary tumour is shown in Table I.

**Table I** The relationship between estimated tumour size (mean±s.e.), clinical stage and survival

| *Group* | *No.* | *Mean tumour volume (ml)* |
|---|---|---|
| Stage I–all | 79 | 97±12 |
| –alive NED | 71 | 96±13 |
| –dead | 5 | 119±30 |
| Stage II–all | 31 | 213±49 |
| –alive NED | 23 | 183±47 |
| –dead | 8 | 300±133 |
| Stage III & IV–all | 7 | 129±24 |

**Table II** The relationship between clinical stage, survival and the interval between first symptom and first treatment (mean±s.e.)

| *Group* | *No.* | *Mean symptom duration (days)* |
|---|---|---|
| Stage I–all | 98 | 199±22 |
| –alive NED | 71 | 96±13 |
| –dead | 5 | 119±30 |
| Stage II–all | 40 | 189±29 |
| –IIA | 20 | 154±22 |
| –IIB, IIC | 20 | 225±51 |
| –alive NED | 30 | 169±22 |
| –dead | 10 | 250±102 |
| Stage III & IV | 8 | 176±87 |

There were no statistically significant differences between the groups.

The average interval between first symptom and first treatment was 198±17 days (range 11–1084). The relationship between duration of symptoms before treatment, survival, and clinical stage is shown in Table II. Once again there were no significant differences between groups. The mean interval between diagnosis and first treatment was 32±2 days.

The relationship between tumour size at diagnosis and duration of symptoms for patients who had symptoms for less than a year before treatment is shown in Figure 5. There were 17 patients who had symptoms for more than a year before treatment. The average size of primary tumour in this group was 224 ml. For patients with clinical stage I seminoma and tumours <75 ml the mean duration of symptoms was 199±31 days; for patients with stage I seminoma and tumours >75 ml the mean duration of symptoms was 256±50 days.

Two patients had elevated levels of $\beta$HCG at diagnosis.

**Figure 5** The relationship between tumour size (mean±s.e.), and duration of symptoms before first treatment.

One patient, clinical stage IIB, remains well after radiotherapy. The other patient who presented with Stage IV disease and $\beta$HCG >100,000 died from respiratory failure 15 days after starting treatment with *cis*-platinum, bleomycin and vinblastine. Only one patient had elevated AFP at diagnosis. This fell, post orchiectomy, with a serum half-life of 6 days. A review of the histology failed to show any features of teratoma. He remains well three years after radiotherapy.

The serum LDH at diagnosis was $321 \pm 17 \mathrm{IU\,l^{-1}}$ in patients with stage I disease and $533 \pm 117 \mathrm{IU\,l^{-1}}$ in patients with stage II disease. LDH was estimated at diagnosis in only two patients with advanced (Stage II or IV) disease, the level was grossly elevated in both patients.

There are few data on tumour marker levels at the time of relapse. None of the four patients who had AFP measured had elevated levels; similarly none of the three patients who had BHCG measured had elevated levels. Six patients had serum LDH measured at relapse: in three the levels were elevated, in three they were normal.

The major morbidity from treatment is summarised in Table III. In addition to the data summarised in Table III the following late complications have been observed. Individual patients treated with abdominal irradiation suffered from: lower limb ischaemia; myocardial infarction; large bowel stricture (post-radiation fibrosis); diverticular disease; gluten sensitive enteropathy; depression/alcohol abuse. Individual patients treated with radiotherapy above and below the diaphragm developed: pulmonary fibrosis; acute cholecystitis; large bowel fibrosis/stricture. As with any analysis of possible complications of treatment it is difficult to be certain which of these various problems can be directly attributed to the radiotherapy.

**Table III** The morbidity from radiotherapy in clinical Stages I and II

| *Complication* | *No.* |
|---|---|
| Abdominal irradiation alone | 111 |
| Early | |
| Marrow depression | 2 |
| Severe nausea and vomiting | 1 |
| Deep venous thrombosis | 1 |
| Late | |
| Duodenal ulcer | 4 |
| Proven infertility | 3 |
| Hypertension | 2 |
| Decreased libido | 2 |
| Chronic diarrhoea | 4 |
| Irradiation above and below the diaphragm | 32 |
| Early | |
| Marrow depression | 4 |
| Dysphagia | 1 |
| Lhermittes sign | 1 |
| Late | |
| Proven infertility | 2 |
| Renal impairment | 2 |

### Discussion

This study, based on patients seen and treated at a regional centre, is free from many of the biases which intrude when the experience of other centres is analysed. Patients are referred to the Edinburgh department simply because of where they live and not because they have advanced disease or present especially difficult problems in management. The low proportion of patients with stage III or IV disease (5.3%) is similar to that in several other large studies (Calman *et al.*, 1979; Schultz *et al.*, 1984; Thomas *et al.*, 1982).

A striking, and gratifying, improvement in prognosis occurred between the earlier and latter years of the study period. The overall actuarial 5-year survival increased from 64.8% to 96.6%. There was no obvious explanation for this change. The introduction of lymphography in 1972–1973 seems, perhaps misleadingly, to have been associated with an improvement in prognosis. Patients assigned to clinical Stage I without having had lymphography had a long term survival rate of 79.2%; patients in clinical Stage I who had lymphography had a long term survival rate of 96.6%. Understaging, due to omission of lymphography, cannot entirely explain the improvement in prognosis since the para-aortic nodes, the site of undetected disease, were irradiated in all patients. Lymphography might have increased the effectivenes of the radiotherapy by improving its localization, but none of the patients who died from Stage I seminoma relapsed in the para-aortic nodes.

Changes in the distribution by stage within the group of patients with seminoma will not improve the survival of the group as a whole; even though they might improve survival within each clinical stage. If, because of some shift in histological criteria, there is a change in the proportion of seminomas in patients with germ-cell tumours then this could affect prognosis for all patients with seminomas (Oliver *et al.*, 1984). In Edinburgh between 1970 and 1973 seminomas accounted for 46/76 (60.5%) of all germ-cell tumours; between 1978 and 1981 52/114 (45.6%) of germ-cell tumours were seminomas (chi-squared = 3.48; $P > 0.05$). Although not achieving statistical significance, this change in histological distribution might account for the improved prognosis after 1978.

The availability of effective therapy for metastatic disease has transformed the overall prognosis for patients with non-seminomatous germ-cell tumours (NSGCT) (Einhorn & Williams, 1980; Newlands *et al.*, 1983). This factor is of less importance for seminomas. Very few patients present with, or develop, haematogenous metastases. Certainly the improved prognosis in Edinburgh cannot be explained by improvements in therapy for disseminated disease: only three patients were treated with *cis*-platinum chemotherapy. Analysis of national data from the USA failed to show any improvement in survival for patients with seminoma between 1973–76 and 1976–79 (Li *et al.*, 1982). The survival rate was 92% during both periods.

The T-stage of the primary tumour affected prognosis: patients with tumours beyond T1 had a significantly lower long term survival (Figure 2). Advanced T-stage of the primary tumour might signal the biologically more aggressive tumours. Unfortunately T-stage takes no account of other possible harbingers of aggressive tumour behaviour such as vascular invasion (Sandeman & Matthews, 1979).

The results of prophylactic radiotherapy for adequately staged patients with clinical Stage I disease are excellent: 96.11% long term survival overall; 100% survival for patients with T1 primary tumours. These results are achieved without excessive morbidity. The relatively high incidence of duodenal ulcer in patients treated for seminoma is puzzling and has been noted by others (Peckham *et al.*, 1985). We have not observed the problem in patients treated with similar radiation fields but higher dose, for non-seminomatous tumours (Duncan & Munro, 1985).

No attempt was made routinely to assess fertility after treatment. A total of 5 patients had proven infertility: the true number is almost certainly higher. The radiation dose to the remaining testis can, by extra shielding, be reduced below the levels achieved in this study (Kubo & Shipley, 1982) with consequent improvement in the fertility rate after treatment.

Second testicular primary tumours were relatively frequent: 4/152 (2.6%). This represents a relative risk of ×500 compared with the normal male population, similar findings have been described in a larger series (Hay *et al.*, 1984). This is in contrast to patients with NSGCT where there is no apparent excess of second testicular tumours (Duncan & Munro, 1985).

The results of radiotherapy for patients in clinical stages II, III and IV are disappointing. The long-term survival rate of 66% for patients with Stage II disease is inferior to that which now can be expected for patients with clinical Stage II NSGCT treated by combination chemotherapy. In common with others (Thomas *et al.*, 1982) we find no evidence to support the concept of prophylactic irradiation above the diaphragm for patients with Stage I or Stage II disease. Radiotherapy to the mediastinum and supraclavicular nodes does not completely prevent relapse at these sites, can only benefit a small minority of patients, and significantly adds to morbidity.

Effective chemotherapy is now available for disseminated seminoma (Schuette *et al.*, 1985; Peckham *et al.*, 1985). In the study from the Royal Marsden Hospital (Peckham *et al.*, 1985) 40/44 (91%) of patients treated with chemotherapy for advanced seminoma are alive and disease-free 12 to 73 months after treatment. This raises the possibility of omitting routine prophylactic radiotherapy to the retroperitoneum for patients with stage I seminoma and relying on chemotherapy to treat those patients who subsequently relapse. If a policy of surveillance after orchiectomy is to be considered for patients with Stage I seminoma then the identification of factors influencing relapse becomes as relevant as finding factors which affect survival.

The relapse rate was significantly higher in clinical Stage II compared with clinical Stage I. Tumour size at diagnosis or duration of symptoms before treatment had little effect on relapse rate. The T-stage of the primary tumour appeared to affect relapse rate but the difference did not quite achieve statistical significance. This is probably due to a Type II statistical error: too few patients at risk in either group.

It is now considered reasonable not to treat the retroperitoneum prophylactically in patients with Stage I NSGCT and enter these patients in surveillance studies (Peckham *et al.*, 1983; Read *et al.*, 1983). The arguments used to support such a policy cannot simply be translated to justify a similar policy for Stage I seminomas. Seminomas are different in their biology and natural history from NSGCT. The most serious defect in any argument in favour of a policy of surveillance for Stage I seminoma is the lack of adequate tumour markers for the tumour. Lactate dehydrogenase levels correlate roughly with the amount of tumour present but the enzyme is in no way a specific monitor of disease activity. Placental alkaline phosphatase, particularly when detected using the monoclonal antibody H17E2, may prove a useful marker for seminoma but raised marker levels in patients who smoke cloud the issue (Tucker *et al.*, 1985; Epenetos *et al.*, 1985).

Data from studies of surveillance in patients with Stage I seminoma are preliminary, the follow-up is short since most of these studies only started in 1982 or 1983. The relapse rate with surveillance for Stage I seminoma is probably between 10 and 15%. Most of the surveillance studies exclude patients with primary tumours that are T3 or T4 and the patients in these studies have usually been staged with lymphography and CT scanning. It is not possible to make a direct comparison between patients in studies of surveillance and all 103 patients with stage I seminoma in the Edinburgh series. The group in the Edinburgh series which most closely approximates the type of patient being entered into studies of surveillance is the group of 38 patients with T1 tumours who had had lymphography. The 5-year survival in this group was 100%; the relapse rate was 2/38 (5.15%). Recent data from Toronto show that patients with Stage I seminoma, whose staging investigations included lymphography and CT scanning, have a relapse rate of 2/150 (1.3%) after prophylactic radiotherapy to the para-aortic nodes (Thomas, 1985).

The data on tumour size at diagnosis (Table I) suggest that patients with Stage II disease have larger primary tumours than patients in clinical stages I, III or IV. It might be that there are two subpopulations of seminomatous tumours. One type is a tumour which metastasizes early, hence the relatively small size of the primary tumour in Stages III and IV. The other type is a tumour which grows slowly and metastasizes later in its course, hence the larger size of primary tumour in Stage II compared to Stage I.

We are unable to confirm the findings of Bosl *et al.* (1981) that the longer the interval between first symptom and diagnosis then the more advanced is the clinical stage of the tumour. Bosl's study included both patients with seminoma and those with non-seminomatous tumours. It may be that delay in treatment only correlates with more advanced disease for patients with non-seminomatous tumours: tumours known to have higher metastatic potential and shorter doubling times than seminomas.

The relationship between tumour size and the duration of symptoms is complex (Figure 4). There is a paradoxical decrease in the size of the primary tumour for patients with symptom duration of more than 110 days before treatment. It is difficult to know whether this apparent decrease is real or artefactual: if it is real it is hard to explain.

The results from Edinburgh show that prophylactic retroperitoneal irradiation, using the fields and dose described, produces excellent survival for properly staged patients with Stage I seminoma. We accept that many patients, who do not have micro-metastatic disease in the retroperitoneum, are being treated unneccessarily. Nevertheless, the morbidity of treatment is low enough, and the overall survival high enough, to justify such a policy. The few patients who relapse can be effectively treated with chemotherapy.

Our current recommendations for the management of patients with seminoma, after inguinal orchiectomy and high cord section, are as follows. Patients in clinical Stage I should routinely have radiotherapy to the retroperitoneal nodes. Patients in clinical Stages IIC, III or IV should be treated with combination chemotherapy using a regime based upon *cis*-platinum. The decision is more difficult for patients in clinical Stage IIA or IIB. Chemotherapy will cure about 90% of such patients (Peckham *et al.*, 1985) but at a high price in terms ot toxicity. Radiotherapy to the retroperitoneal nodes will cure approximately 80% of such patients and its toxicity is less than that of chemotherapy. The 20% who are not cured by radiation therapy will require salvage chemotherapy. On balance, a policy of initial treatment with radiotherapy can be justified for patients with Stage IIA or IIB disease; as chemotherapy for seminoma becomes less toxic this balance may change in favour of initial treatment with cytotoxic drugs.

We thank the surgeons and radiotherapists of the Edinburgh region for referring patients; the departments of diagnostic radiology at the Royal Infirmary, Edinburgh and the Western General Hospital, Edinburgh for their help with the staging investigations; the medical records staff in the department of Radiation Oncology, Western General Hospital for their invaluable assistance in locating case-notes and ensuring adequate follow-up of the patients.

### References

BALL, D., BARRETT, A. & PECKHAM, M.J. (1982). The management of metastatic seminoma testis. *Cancer*, **50**, 2289.

BOSL, G.J., GELLER, N.L., CIRRINCIONE, C. & 4 others (1983). Serum tumour markers in patients with metastatic germ cell tumours of the testis. A 10-year experience. *Amer. J. Med.*, **75**, 29.

CALMAN, F.M.B., PECKHAM, M.J. & HENDRY, W.J. (1979). The pattern of spread and treatment of metastases in testicular seminoma. *Br. J. Urol.*, **51**, 154.

DUNCAN, W. & MUNRO, A.J. (1985). The management of early stage non-seminomatous germ cell tumours of the testis: Edinburgh 1970–1981. *Br. J. Urol.*, **57**, 560.

EINHORN, L.H. & WILLIAMS, S.D. (1980). Chemotherapy of disseminated testicular cancer. A random prospective study. *Cancer*, **46**, 1339.

EPENETOS, A.A., MUNRO, A.J., TUCKER, D.F. & 6 others (1985). Monoclonal antibody assay of serum placental alkaline phosphatase in the monitoring of testicular tumours. *Br. J. Cancer*, **51**, 641.

HAY, J.H., DUNCAN, W. & KERR, G.R. (1984). Subsequent malignancies in patients irradiated for testicular tumours. *Br. J. Radiol.*, **57**, 597.

KUBO, H. & SHIPLEY, W.U. (1982). Reduction of the scatter dose to the testicle outside the radiation treatment fields. *Int. J. Radiat. Oncol. Biol. Phys.*, **8**, 1741.

LI, F.P., CONNELLY, R.R. & MYERS, M. (1982). Improved survival rates among testis cancer patients in the United States. *JAMA*, **247**, 825.

NEWLANDS, E.S., BEGENT, R.H.J., RUSTIN, G.J.S. & 2 others (1983). Further advances in the management of malignant teratomas of the testis and other sites. *Lancet*, **i**, 948.

OLIVER, R.T.D., HOPE-STONE, H.F., BLANDY, J.P. (1984). Possible new approaches to the management of seminoma of the testis. *Br. J. Urol.*, **56**, 729.

PECKHAM, M.J., BARRETT, A., McELWAIN, T.J. & 2 others (1981). Non-seminoma germ cell tumours (malignant teratoma) of the testis. Results of treatment and analysis of prognostic factors. *Br. J. Urol.*, **53**, 162.

PECKHAM, M.J., BARRETT, A., HORWICH, A. & 1 other (1983). Orchiectomy alone for Stage I testicular non-seminoma. *Br. J. Urol.*, **55**, 754.

PECKHAM, M.J., HORWICH, A. & HENDRY, W.F. (1985). Surveillance following orchidectomy for clinical stage I testicular germ cell malignancy, In *Germ Cell Tumours II* (Advances in the Biosciences volume **55**) (eds) Jones, W.G. *et al.*, p. 441. Pergamon Press: Oxford.

READ, G., JOHNSON, R.J., WILKINSON, P.M. & EDDLESTON, B. (1983). Prospective study of follow-up alone in Stage I teratoma of the testis. *Br. Med. J.*, **287**, 1503.

READ, G., ROBERTSON, A.G. & BLAIR, V. (1983). Radiotherapy in seminoma of the testis. *Clin. Radiol.*, **34**, 469.

SANDEMAN, T.F. & MATTHEWS, J. (1979). The staging of testicular tumours. *Cancer*, **43**, 2514.

SCHUETTE, J., NIEDERLE, N., SCHEULEN, M.E., SEEBER, S. & SCHMIDT, C.G. (1985). Chemotherapy of metastatic seminoma. *Br. J. Cancer*, **51**, 467.

SCHULTZ, H.P., VON DER MASSE, H., RORTH, M., PEDERSEN, M., SANDBERG NIELSEN, E. & WALBOM-JØRGENSEN, S. (1984). Testicular seminoma in Denmark 1976–1980. Results of treatment. *Acta. Radiol. Oncol.*, **23**, 263.

THACKRAY, A.C. & CRANE, W.A.J. (1976). Seminoma. In *Pathology of the Testis*, (ed) Pugh, R.C.B. p. 164. Oxford: Blackwell.

THOMAS, G.M., RIDER, W.D., DEMBO, A.J. & 5 others (1982). Seminoma of the testis: results of treatment and patterns of failure after radiation therapy. *Int. J. Radiat. Oncol. Biol. Phys.*, **8**, 165.

THOMAS, G.M. (1985). Controversies in the management of testicular seminoma. *Cancer*, **55**, 2296.

TUCKER, D.F., OLIVER, R.T.D., TRAVERS, P. & BODMER, W.F. (1985). Serum marker potential of placental alkaline phosphatase-like activity in testicular germ cell tumours evaluated by H17E2 monoclonal antibody assay. *Br. J. Cancer*, **51**, 631.

UNION INTERNATIONALE CONTRE LE CANCER (1978). *TNM Classification of Malignant Tumours*. Third edition. International Union against Cancer: Geneva.